# MECHANISMS GENERATING CANCER GENOME COMPLEXITY FROM A SINGLE CELL DIVISION ERROR

**DOI:** 10.1126/science.aba0712

**Published:** 2020-04-17

**Authors:** Neil T. Umbreit, Cheng-Zhong Zhang, Luke D. Lynch, Logan J. Blaine, Anna M. Cheng, Richard Tourdot, Lili Sun, Hannah F. Almubarak, Kim Judge, Thomas J. Mitchell, Alexander Spektor, David Pellman

**Affiliations:** 1. Howard Hughes Medical Institute, Chevy Chase, MD, USA; 2. Department of Cell Biology, Harvard Medical School, Boston, MA, USA; 3. Department of Pediatric Oncology, Dana-Farber Cancer Institute, Boston, MA, USA; 4. Department of Biomedical Informatics, Harvard Medical School, Boston, MA, USA; 5. Department of Data Sciences, Dana-Farber Cancer Institute, Boston, MA, USA; 6. Wellcome Sanger Institute, Hinxton, Cambridgeshire, CB10 1SA, UK; 7. Cambridge University Hospitals NHS Foundation Trust, Cambridge, CB2 0QQ, UK; 8. Single-Cell Sequencing Program, Dana-Farber Cancer Institute, Boston, MA, USA; 9. Department of Radiation Oncology, Dana-Farber Cancer Institute, Boston, MA, USA

## Abstract

The chromosome breakage-fusion-bridge (BFB) cycle is a mutational process that produces gene amplification and genome instability. Signatures of BFB cycles can be observed in cancer genomes alongside chromothripsis, another catastrophic mutational phenomenon. Here, we explain this association by elucidating a mutational cascade that is triggered by a single cell division error—chromosome bridge formation—that rapidly increases genomic complexity. We show that actomyosin forces are required for initial bridge breakage, following which chromothripsis accumulates beginning with aberrant interphase replication of bridge DNA. This is then followed by an unexpected burst of DNA replication in the next mitosis, generating extensive DNA damage. During this second cell division, broken bridge chromosomes frequently mis-segregate and form micronuclei, promoting additional chromothripsis. We further show that iterations of this mutational cascade generate the continuing evolution and sub-clonal heterogeneity characteristic of many human cancers.

## INTRODUCTION

Cancer genomes can contain hundreds of chromosomal rearrangements (1). Traditionally, it was assumed that these genomes evolve gradually by accruing small-scale changes successively over many generations. However, the high number of rearrangements in many cancers suggests a non-exclusive, alternative view: cancer genomes may evolve rapidly via discrete episodes that generate bursts of genomic alterations (1–8). This model is appealing because a small number of catastrophic mutational events can parsimoniously explain the origin of extreme complexity in many cancer genomes (4).

At least three classes of catastrophic events may account for a substantial fraction of chromosome alterations in cancer: whole-genome duplication, chromothripsis, and chromosome breakage-fusion-bridge cycles. The first class, whole-genome duplication, can promote tumorigenesis (3) and is now appreciated to occur during the development of ~40% of human solid tumors (9).

The second class, chromothripsis, is extensive rearrangement of only one or a few chromosomes, generating a characteristic DNA copy number pattern (4, 6, 10). Chromothripsis occurs with frequencies of 20–65% in many common tumor types (1, 2, 11). We previously determined that chromothripsis can originate from micronuclei, which arise from mitotic segregation errors or unrepaired DNA breaks that generate acentric chromosome fragments (12–15). Due to aberrant nuclear envelope assembly around these chromosomes, micronuclei undergo defective DNA replication and spontaneous loss of nuclear envelope integrity, which results in extensive DNA damage by unknown mechanisms (16, 17).

The third class of catastrophic event, the chromosome breakage-fusion-bridge (BFB) cycle (18, 19), starts with the formation of another abnormal nuclear structure, a chromosome bridge. Bridges arise from end-to-end chromosome fusions after DNA breakage or telomere crisis, incomplete DNA replication, or failed resolution of chromosome catenation (20). Bridge breakage then initiates a process that can generate gene amplification over multiple cell generations. Although BFB cycles are a major source of genome instability, the sequence pattern of consecutive foldback rearrangements expected from the original BFB model is not commonly observed in cancer genomes without other chromosome alterations (1, 11, 21). Whether subsequent chromosomal rearrangement obscures the simple BFB pattern, or whether the BFB process itself is inherently more complex than originally envisioned has been unclear. Recently, examples of cancer genomes where BFB cycle are intermingled with chromothripsis were identified, raising the possibility that BFB cycles and chromothripsis might be mechanistically related (21–23).

Determining the generality of the association between chromothripsis and BFB cycles requires detailed mechanisms for each step in the BFB cycle, particularly how chromosome bridges are broken. Proposed mechanisms for chromosome bridge breakage have included breakage by spindle forces during the mitosis in which they are formed or DNA cleavage by the cytokinesis/abscission apparatus (19, 24–26). Yet recent work indicates that breakage of chromosome bridges, at least the “bulky” bridges visible by DNA staining (27), is uncommon during mitosis or cytokinesis and they instead persist for many hours into interphase (28, 29). It was then proposed that interphase bridges are severed by the cytoplasmic, endoplasmic reticulum-associated exonuclease, TREX1 (28). Transient nuclear envelope (NE) disruption was suggested to allow TREX1 to enter the nucleus, where it could simultaneously break the bridge and fragment bridge DNA to generate chromothripsis (28). Although the TREX1 model can explain the association between BFB cycles and chromothripsis in cancer genomes (21), loss of TREX1 was reported to delay, but not block, bridge breakage (28).

Below, we present data supporting a new model for the genomic consequences of BFB cycles, explaining its association with chromothripsis. Rather than being generated simultaneously by a single mechanism, we demonstrate that chromothripsis accumulates through a cascade of mutational events initiated by the formation of a chromosome bridge. We observed an analogous series of events after the formation of micronuclei, suggesting a unifying model for how cancer-associated defects in nuclear architecture (“nuclear atypia”) promote genome instability. Together, these findings reveal how a single cell division error rapidly generates extreme genomic complexity and continually evolving subclonal heterogeneity.

## RESULTS

**Figure 1.**
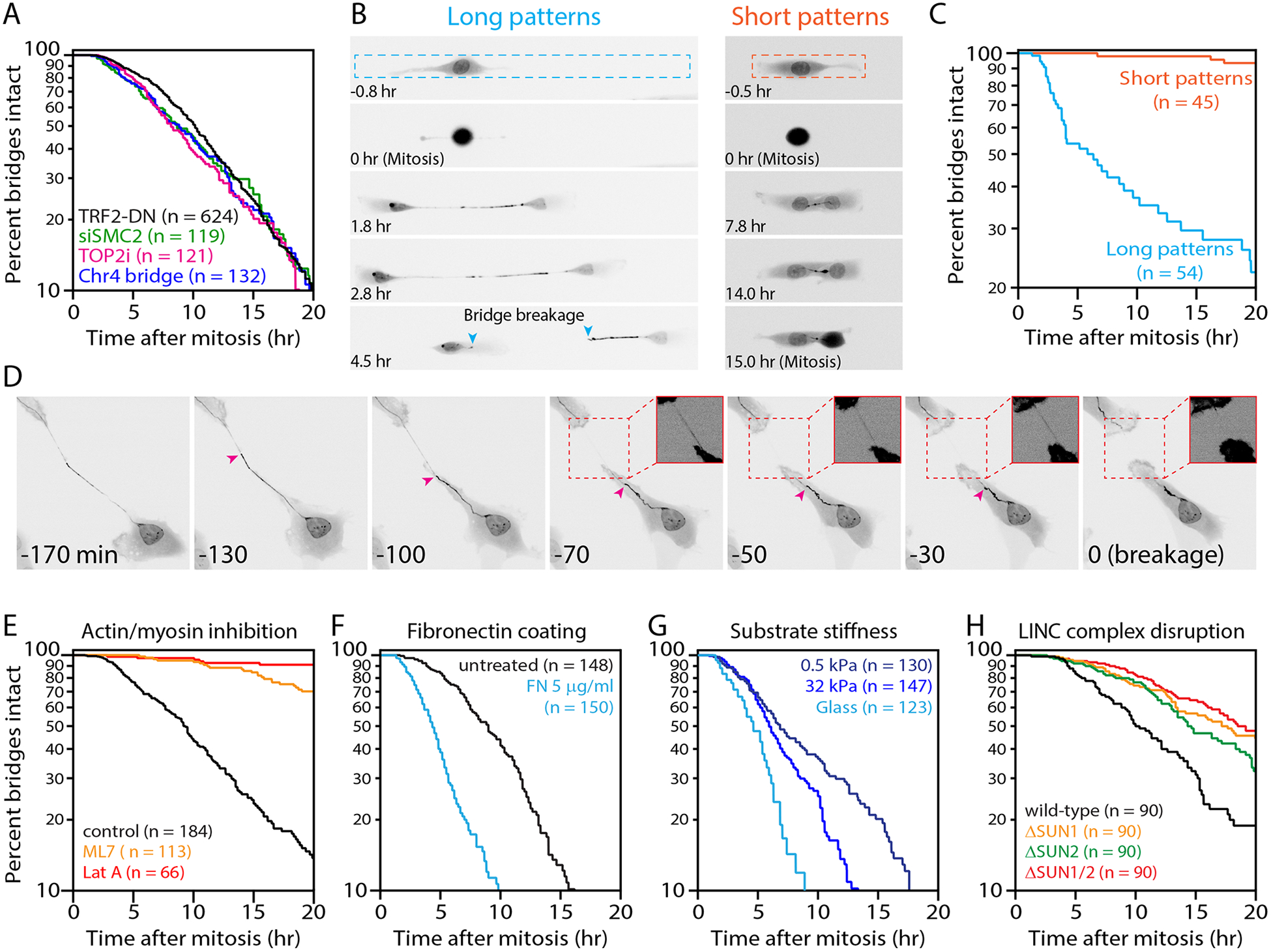
Chromosome bridge breakage requires actomyosin contractility. (A) Indistinguishable chromosome bridge lifetimes observed with different methods for bridge induction. Shown are bridge lifetimes (time from bridge formation until breakage or mitotic entry). Bridges were visualized with GFP-BAF and generated by: inducible TRF2-DN (n=624 bridges analyzed), condensin partial knockdown (siSMC2, n=119), low-dose ICRF-193 (n=121), or inducible CRISPR/Cas9 cutting of Chr4 subtelomere (n=132). These mean bridge lifetimes are not significantly different (*p*=0.14, one-way ANOVA). (B) Extension of chromosome bridges is required for their breakage. Time-lapse images (GFP-BAF) of cells with bridges on “long” (20×300 μm) or “short” (20×100 μm) fibronectin micropatterns. Bridge length does not exceed ~50 μm on short patterns. Dashed lines: micropattern borders. Teal arrowheads: broken bridge ends. Timestamp is relative to completion of the previous mitosis. (C) Quantification from (B): bridge lifetime on short (n=45) and long (n=54) micropatterns (*p*<0.0001, Mann-Whitney). (D) Representative chromosome bridge breakage event. Prior to breakage, there is apparent non-uniform stretching of the bridge (GFP-BAF). Magenta arrowhead: a transition between “taut” and “slack” regions of the bridge. The taut region progressively stretches, the slack region progressively retracts, and breakage occurs in the taut region. Inset images: high contrast of the taut region (dashed red boxes) before and after breakage. Timestamp is relative to bridge breakage. (E) Actomyosin contractility is required for bridge breakage. Cells were allowed to divide and form bridges before exchange into drug medium (see Fig. S5D for scheme). Plot shows bridge lifetimes with actin disruption (LatA, n=66), myosin-II inhibition (ML7, n=113), and control (DMSO, n=184). (F) Increased cellular contractility decreases bridge lifetime. Bridge lifetimes on untreated glass (n=148) or fibronectin (FN)-coated glass (n=150). (G) Bridge breakage timing depends on substrate stiffness: glass (>10^6^ kPa, n=123), stiff gel (32 kPa, n=147), and soft gel (0.5 kPa, n=130). All substrates were coated with 5 μg/ml fibronectin. (H) Partial requirement of LINC complex for bridge breakage: wild-type (n=90), ΔSUN1 (n=90), ΔSUN2 (n=90), and ΔSUN1/ΔSUN2 (n=90) RPE-1 cells.

Four methods were used to generate chromosome bridges: transient expression of a dominant negative variant of telomeric repeat-binding factor 2 (TRF2-DN) (30), partial knockdown of condensin (siSMC2) (31), low-dose topoisomerase II inhibition (ICRF-193) (32), and CRISPR/Cas9-mediated telomere loss on chromosome 4 (Chr4 bridge, Fig. S1A–C; a list of bridge-induction methods for each experiment is provided in Table S1). Chromosome bridges were visualized in live cells with GFP-BAF [barrier-to-autointegration factor (33)], a sensitive reporter for these structures whose signal is not compromised by stretching of the bridge (Fig. 1), unlike fluorescent histone reporters (28). For TRF2-DN, we developed conditions for transient expression and live-cell imaging that avert the previously reported strong inhibition of cell cycle progression (28). In our conditions, cells with bridges entered S phase with similar timing after mitotic exit as unperturbed cells lacking bridges (8.3 versus 7.3 hr, respectively; Fig. S1D and accompanying legend). Importantly, bridges generated by these different methods had similar median lifetimes (t_1/2_): ~10 hours from the completion of mitosis (Fig. 1A).

### Mechanical force triggers chromosome bridge breakage

The TREX1 exonuclease was proposed to cleave chromosome bridges after rupture of the primary nucleus (28). However, we did not detect a delay in the timing of bridge breakage in TREX1 knockout cells, even when using the same cell lines and bridge induction method (28) (in total, two different bridge induction methods were employed, and six independent clones from two knockout strategies were tested; Fig. S2A–C). Additionally, 36% of bridge breakage events occurred in the absence of detectable rupture of the primary nucleus (n=58), and bridge lifetime showed no correlation with the duration of nuclear envelope disruption (Fig. S2D). These findings suggested that fundamental aspects of the mechanism for bridge breakage remained to be identified.

A clue for alternative mechanisms to TREX1 came from the observation that bridges can reach hundreds of microns in length before breaking as interphase cells migrate in culture, suggesting that bridge breakage might have a mechanical component. Accordingly, we found that motile cell lines broke bridges during interphase with similar timing, whereas bridges in less motile cell lines almost never extended beyond 100 μm and rarely underwent breakage before the next mitosis (Fig. S3).

To determine if bridge extension is required for breakage, we constrained cell migration and bridge extension using rectangular fibronectin “micropatterns” (34). When RPE-1 cells were plated on long (300 μm) patterns, newly formed chromosome bridges extended to ~160 μm on average and broke during interphase with similar kinetics as in unconstrained cells (Fig. 1B–C, Movie S1). By contrast, restricting bridge extension with short (100 μm) micropatterns limited bridge extension to <50 μm and almost completely blocked bridge breakage (<10% bridge cleavage prior to entry into the next mitosis; Fig. 1B–C, Movie S2). There was also less spontaneous NE rupture on short pattern, but increasing NE ruptures >8-fold with Lamin B1 knockdown failed to accelerate bridge breakage (Fig. S4). Therefore, the extension of chromosome bridges, but not NE rupture, is required for their breakage.

Mechanical forces could stretch a bridge across its length or act locally within a section of a bridge. Consistent with the latter model, bridges often formed acute angle bends and/or exhibited non-uniform stretching prior to breakage, with one segment appearing taut and adjacent segments appearing slack, followed by breakage within the taut segment (23 of 25 cases examined, Fig. 1D, Movie S3). Moreover, live-cell imaging revealed the accumulation of large concentrations of actin filaments immediately adjacent to the taut segments of the bridge just prior to breakage in all cases examined (n=30; Fig. S5A, Movie S4 and see (29) for similar results). Actin accumulations were transient and dissolved after bridge breakage. Large focal adhesions and active, phosphorylated myosin II were also observed at these sites (Fig. S5B–C), consistent with local myosin accumulation and high contractility induced by increased membrane tension (35) and indicating strong cell attachments to the extracellular matrix.

To determine if actomyosin contractility is required for chromosome bridge breakage, chromosome bridges were generated, allowed to extend, and exposed to small-molecule inhibitors of myosin activation (ML7) or actin assembly (Latrunculin A). ML7 addition substantially delayed, and Latrunculin A addition abolished, bridge breakage (Fig. 1E, Fig. S5D, and Movie S5). We note that although LatA blocks cell motility and thus prevents further bridge extension after drug addition, ML7 treatment did not have a significant effect on bridge extension (bridge length before breakage or entry into the next mitosis, mean ± SEM: ML7, 140 ± 11 μm, DMSO, 150 +/−12 μm; p=0.56, Mann-Whitney test). Therefore, the prolonged bridge lifetime in ML7-treated cells cannot be explained by an inability to extend bridges. These findings demonstrate that a functional actomyosin network is essential for bridge breakage. Moreover, when cells were plated on fibronectin, which increases focal adhesions and intracellular actomyosin contractile forces (36), bridge breakage was accelerated two-fold (*p*<0.0001; Fig 1F). Because fibronectin also affects cell signaling (37), we plated cells on hydrogels of varying stiffness, all coated with the same concentration of fibronectin. Consistent with reduced substrate stiffness causing diminished actomyosin contractility (38), bridge lifetime was prolonged on softer substrates (Fig. 1G). Finally, knockout of SUN1 and SUN2, the major inner nuclear membrane LINC components that transmit actomyosin forces across the nuclear envelope (39), caused a partial delay in bridge breakage (t_1/2_=18 hr; Fig 1H and Fig. S6). Together, these data establish a critical role for cytoplasmic actomyosin contractile forces in chromosome bridge breakage.

### Single-cell sequencing to determine the immediate impact of chromosome bridge breakage

#### Copy number alterations immediately after bridge breakage.

**Figure 2.**
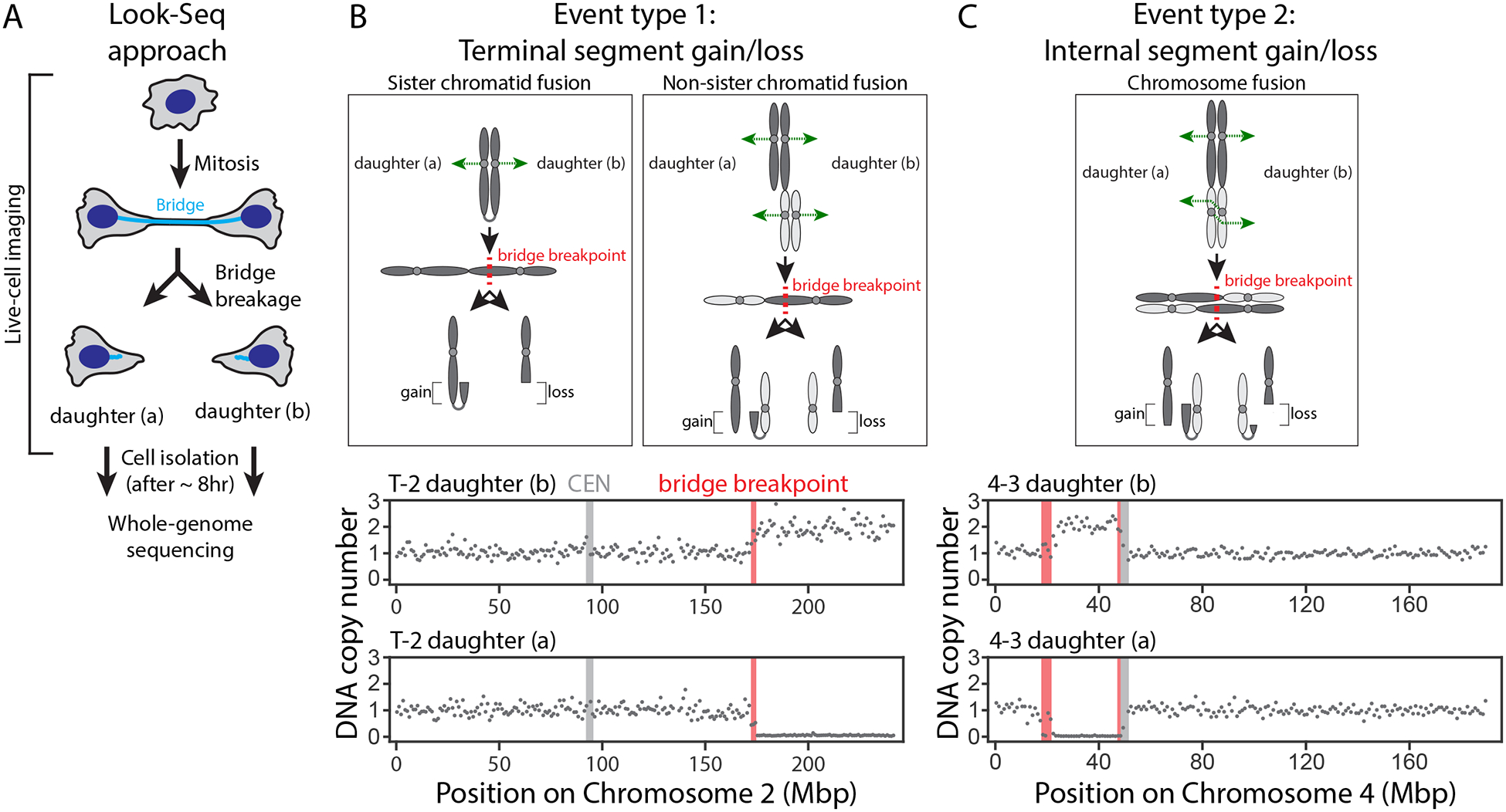
Immediate effect of chromosome bridge breakage on DNA copy number. (A) Cartoon illustrating Look-Seq experiments. (B) Type 1 events are daughter cells with reciprocal gain and loss of a terminal chromosome segment. **Top**: Sister (left) and Non-sister (right) chromatid fusions. In mitosis, the resulting dicentrics are segregated (green dashed arrows), forming a bridge. Bridge breakage (dashed red line) produces copy number alterations as shown. **Bottom**: representative copy number plot (gray dots, 1-Mb bins for the affected Chr2 haplotype). Red bar: inferred bridge breakpoint. Light gray bar: centromere. (C) Type 2 events are reciprocal gain and loss of an internal chromosome segment between the daughter cells. **Top**: a chromosome fusion (40). If the kinetochores of each dicentric attach to microtubules from opposite poles as shown (dashed green arrows), the dicentric chromatids invert relative to each other. Cleavage of the antiparallel chromatid pair yields reciprocal copy number alterations of an internal chromosome segment. **Bottom**: DNA copy number plot as in (B).

To identify the immediate outcome(s) of bridge breakage without confounding genomic alterations during subsequent cell divisions, we employed a combination of live-cell imaging with single-cell whole-genome sequencing [Look-Seq (15)]. Chromosome bridges were induced, their breakage was monitored, and the two daughter cells were isolated ~8 hr after bridge breakage for sequencing (Fig. 2A). Sequencing was performed to ~25× genome coverage, covering ~90% of the unique sequence of each homologous chromosome with one or more reads (Supplemental Methods).

The BFB model (19) predicts that daughter cells should exhibit reciprocal terminal chromosome segment gain and loss patterns due to breakage of dicentric fusions of sister chromatids or single chromatids from different chromosomes (“chromatid-type fusions,” Fig. 2B). Indeed, in all 20 daughter cell pairs after bridge breakage, we observed reciprocal exchange affecting a segment (>2.5 Mb) of one or more chromosome arms (Fig. S7). Using previously developed haplotype copy number analysis (15), we could unambiguously identify the homologous chromosome(s) that underwent breakage. Unexpectedly, in four daughter cell pairs, we observed the reciprocal gain and loss of internal chromosome segments. This pattern can be explained by bridge breakage when a pair of dicentric fusions composed of the replicated chromatids from two different chromosomes is formed (“chromosome-type fusions,” (40), Fig. 2C). In this circumstance, internal chromosome segment exchange results if kinetochore-microtubule attachments occur in a way that generates an antiparallel orientation of the paired dicentrics. We note that without information from both daughter cells, the internal segment gain in one daughter could be misinterpreted as replication-based sequence duplication, rather than chromosome breakage (see Supplemental Text). Although bridge breakage sometimes affected only one chromosome, in 9 cases, two or more different chromosomes were involved, as expected from the methods employed to induce bridges (41); the exception was the CRISPR-based method, which exclusively produced chromosome 4 bridges as expected (Fig. S7).

**Figure 3.**
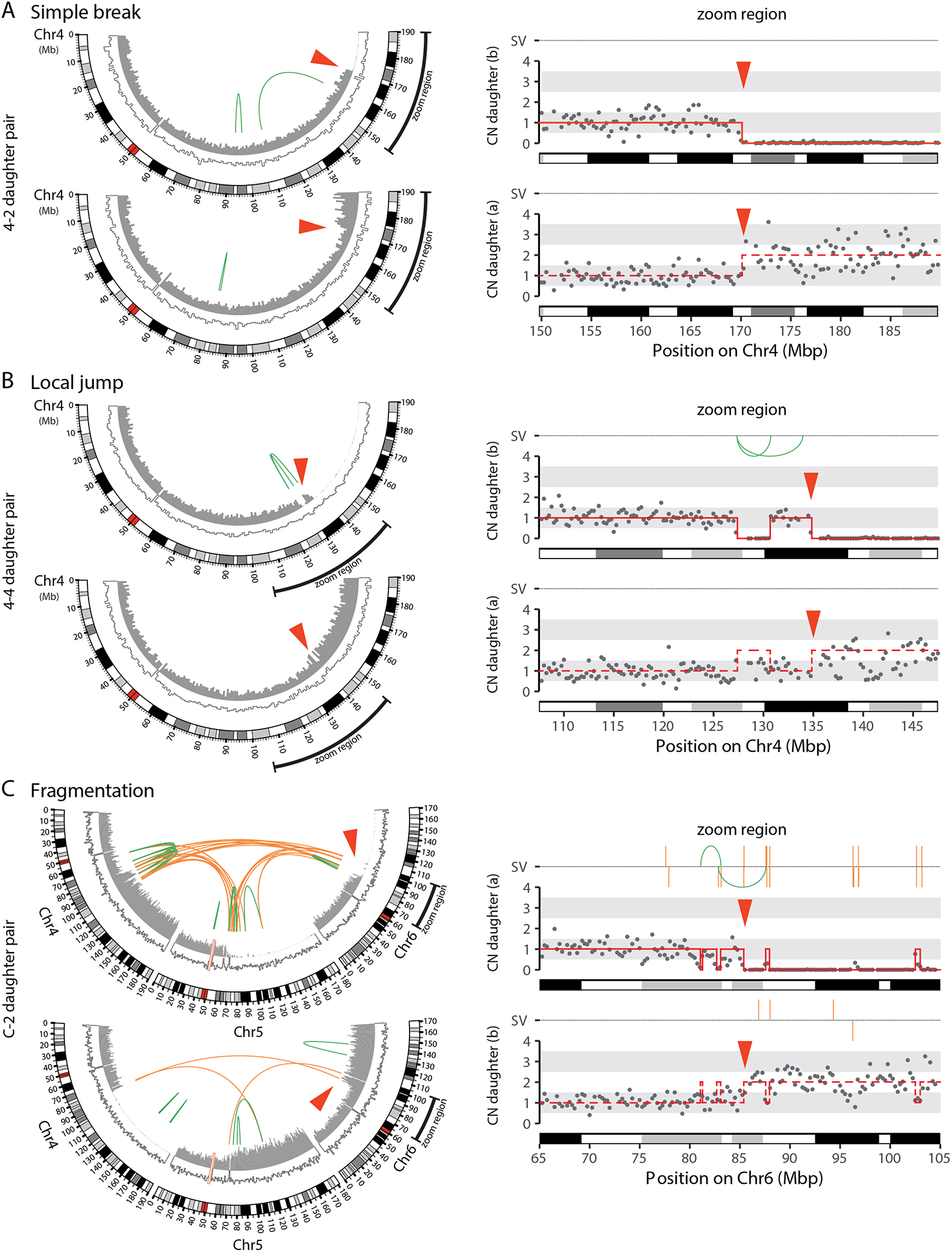
Localized DNA breakage and rearrangement with bridge breakage. (A) Simple bridge breakage. **Left**: CIRCOS plots showing the bridge chromosome (Chr4). Outer arc: chromosome cytoband. Inner arcs: DNA copy number for the bridge haplotype (filled gray bars) and the non-bridge haplotype (white bars, gray outline). Green lines: intrachromosomal structural variants (SVs). Red arrowhead: bridge breakpoint. **Right**: Zoom-region plot shows copy number (gray dots: 250-kb bins) near the bridge breakpoint. Copy-number segments (red solid lines) were determined using SNP-level coverage in the top daughter (Supplemental Methods); the bottom daughter is inferred to contain reciprocal copy-number segments (dashed red lines). SVs, as in CIRCOS plots, are shown above. (B) Bridge breakage can produce the “local jump” pattern. As in (A), CIRCOS plots (Left) and zoom-region plot (Right) for the bridge chromosome (Chr4). (C) Local fragmentation and complex rearrangement with bridge breakage. As in (A), CIRCOS plots (Left) and zoom-region plot (Right) for a bridge containing three different chromosomes (Chr4, Chr5, and Chr6) showing local fragmentation. The pattern of rearrangements in daughter (b) indicates end-joining of these fragments, producing intra- and inter-chromosomal rearrangements (green and orange lines, respectively). Daughter (a) additionally evidences the TST jump rearrangement pattern (see Fig. 5).

Closer inspection of the bridge breakpoints revealed a spectrum of copy number alterations near the break: some bridges underwent simple breakage, and others experienced fragmentation localized to the region of the main copy number transition (Fig. 3). In cases where bridge breakage occurred with local fragmentation, fragments as small as ~100 kb could be detected with confidence if these fragments were retained within a larger region of complete haplotype loss. Rearrangements involving fragment ends often provided additional support for these copy number alterations (Supplemental Methods and see below).

**Figure 4.**
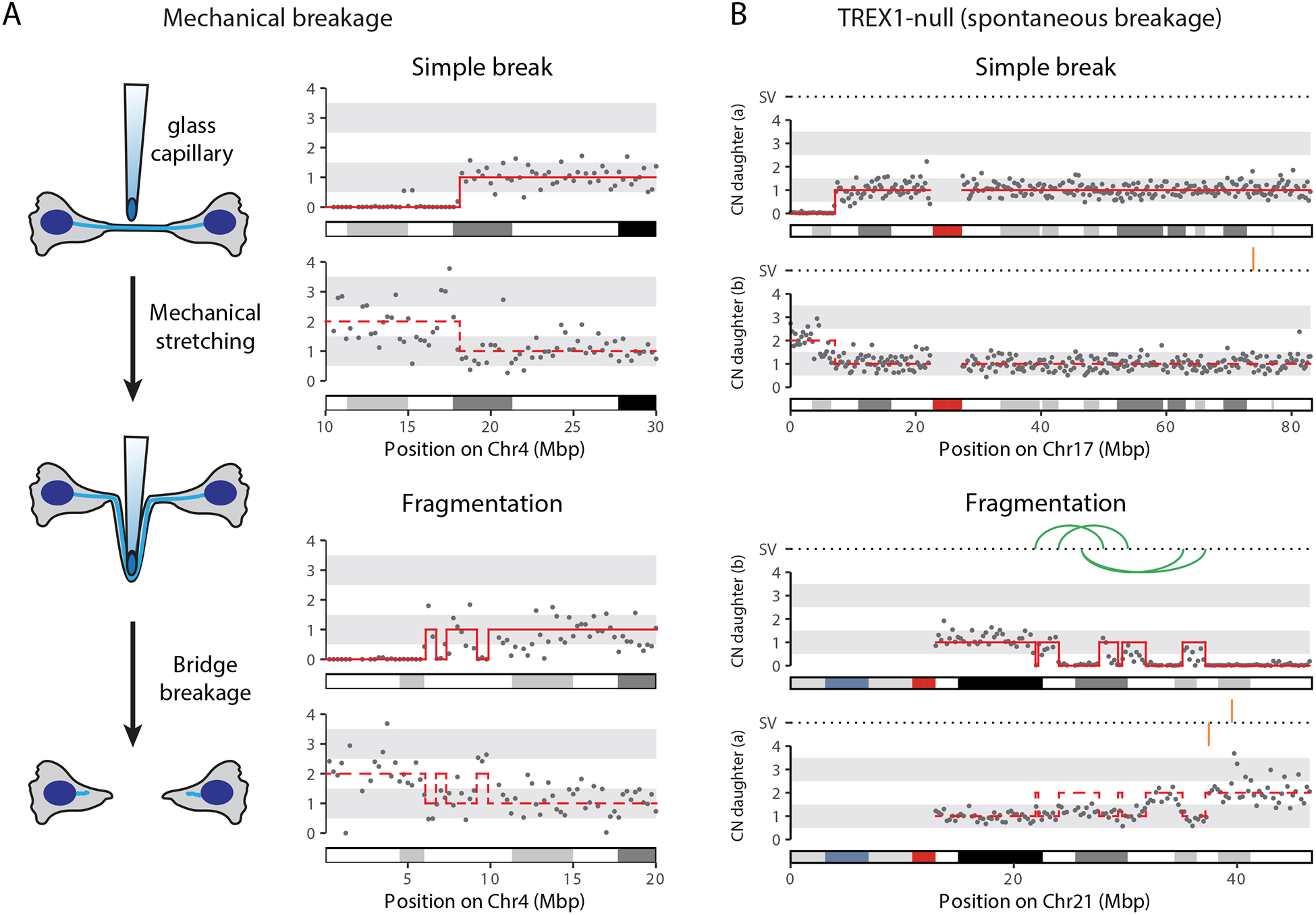
Local fragmentation accompanies mechanical bridge breakage and does not require TREX1. (A) Mechanical bridge breakage produces simple breaks and local fragmentation. **Left**: schematic of the experiment. Cells were collected immediately after mechanical bridge breakage to determine its direct consequences (i.e. not allowing time to generate chromosomal rearrangements). **Right**: Copy number plots, as in Fig. 3, show examples of simple bridge breakage (top) and local fragmentation (bottom). (B) Copy number and SV plots, in Fig. 3: simple bridge breakage (top) and local fragmentation (bottom) after spontaneous bridge breakage in TREX1-null cells.

To determine if simple breaks and local fragmentation can be directly generated by mechanical force, we used a glass capillary to mechanically break chromosome bridges (Movie S6). Strikingly, this yielded both simple breaks and local fragmentation (Fig. 4A and Fig. S8A). Moreover, we observed similar local fragmentation patterns for spontaneous bridge breakage in TREX1-null cells, reinforcing the conclusion that TREX1 is not required to break or fragment chromosome bridges (Fig. 4B and Fig. S8B).

In summary, these findings demonstrate that the immediate genomic consequences of bridge breakage are relatively simple patterns of copy number alterations localized near the site(s) of breakage on bridge chromosomes. This localized pattern contrasts with what is observed in bulk populations of cells isolated many generations after telomere crisis; these populations often contained complex copy number alterations and rearrangements that encompassed most of a chromosome arm and/or spanned the centromere (28). We observed similar complex patterns in long-term population evolution experiments and will present evidence defining a cascade of events downstream of initial bridge breakage that can explain them (see Figs. 6–7 and Fig. S23 below).

#### Chromosome rearrangements associated with bridge breakage.

We next analyzed chromosome rearrangements associated with the above-described DNA copy number alterations. Many cell pairs exhibiting local fragmentation contained rearrangements expected from ligation of the fragments (Fig. 3B). In some cases, local fragmentation affected two or more bridge chromosomes, leading to both inter- and intra-chromosomal rearrangements (Fig. 3C, bottom cell). This pattern of rearrangements resembles the “local n-jump” or “local-distant” rearrangement clusters identified from a recent analysis of structural variation in cancer genomes (42). Therefore, at least some these patterns (hereafter “local jumps”) likely occur by chromosome fragmentation and DNA ligation, in common with rearrangements meeting conventional criteria for chromothripsis. Thus, consistent with our previous proposal, the mechanisms generating chromothripsis can also produce less extreme outcomes, suggesting that the frequency of chromothripsis-like phenomena in cancer genomes may be underestimated (15).

#### Tandem Short Template (TST) jumps.

**Figure 5.**
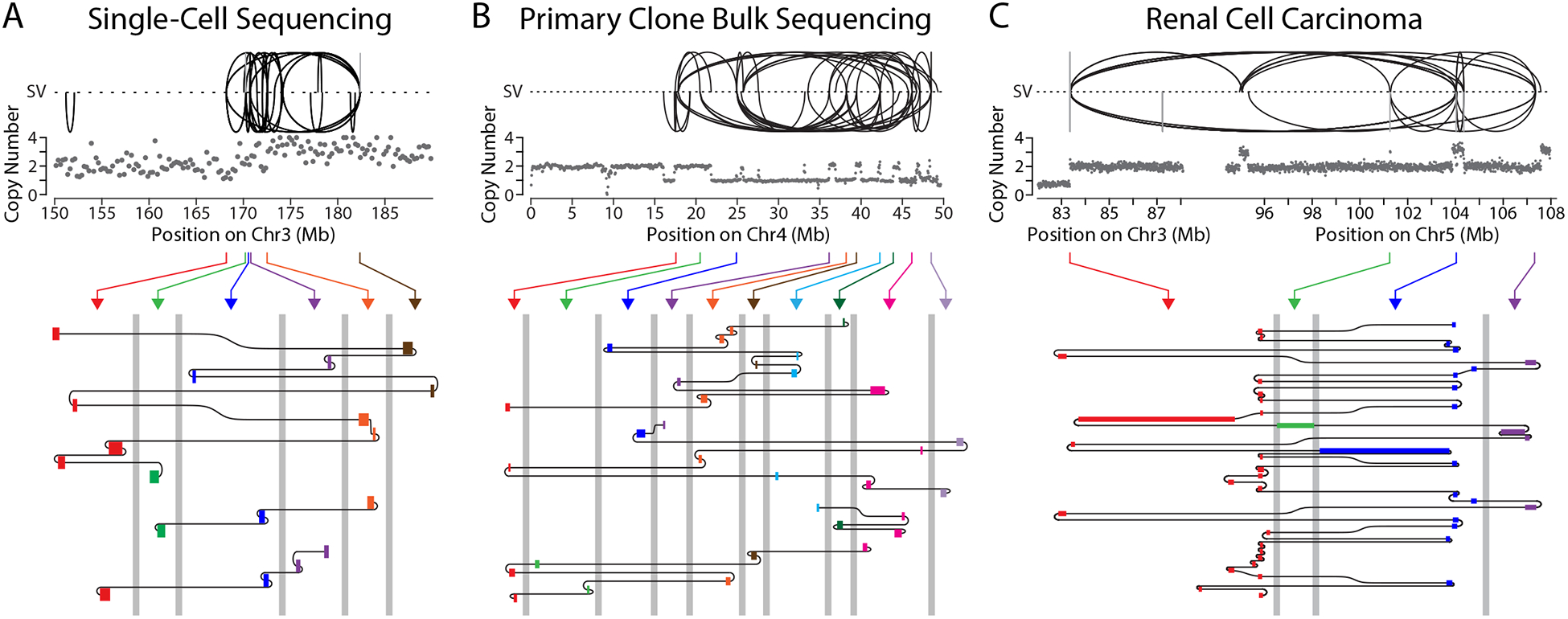
The Tandem Short Template (TST) jump rearrangement signature. (A) Features of the TST jump signature. **Top**: plots show copy number (gray dots, 250-kb bins) and SVs (black: intrachromosomal; gray interchromosomal) for a region near the bridge breakpoint on Chr3. **Bottom**: schematic shows three chains of templated insertions (rectangles), colored according to their origin from six breakpoint hotspots (arrows from Top). Templated insertions are connected as shown by black lines, in a zoom-region view for each breakpoint hotspot (≤10-kb window in each region). Grey vertical lines are axis breaks indicating distances of >10 kb between the hotspots. (B) The TST jump signature in bulk sequencing data from a primary clone after bridge breakage. As in (A), **Top**: copy number (250-kb bins) and SVs for the bridge chromosome (Chr4). **Bottom**: four chains of templated insertions originating from 10 breakpoint hotspots. (C) TST jump signature in long-read sequencing from a renal cell carcinoma sample. As in (A), **Top**: copy number (10-kb bins) and SVs for the region of unbalanced translocation between Chr3 and Chr5. **Bottom**: one chain of templated insertions originating from four breakpoint hotspots (3- to 10-kb windows).

Four daughter cell pairs showed a distinct and particularly striking pattern of complex rearrangement (n=4 of 20 cell pairs; Fig. 5A). Additionally, two of these cases also evidenced kataegis, a phenomenon in which local clusters of point mutations are generated by APOBEC family cytosine deaminases on single-stranded DNA (Fig. S9) (28, 43, 44). The rearrangement junctions from these four samples had features that are inconsistent with an origin from simple fragmentation followed by ligation in random order and orientation. Instead, this pattern suggests an origin from errors during DNA replication. First, rather than being randomly distributed, breakpoints were tightly clustered into local 1–10 kb hotspots (Fig. S10A). Second, tracking the connections between rearrangements revealed chains of tandemly arrayed short insertions (median insertion size 183 bp; Fig. S10B), which we refer to as “Tandem Short Template” (TST) jumps (Fig. 5). The TST insertions typically originated from break ends generated by local fragmentation within the bridge, but were occasionally derived from intact chromosomes not obviously in the bridge.

It is likely that TST jumps were generated by template-switching errors in DNA replication, as in the microhomology-mediated break-induced replication (MMBIR) model (10, 45). Accordingly, we analyzed microhomology at the junctions between TST insertions. Although a minority of junctions showed blunt end-joining, junctions with obvious microhomology were also infrequent. For example, of the 13 junctions in one TST chain shown in Fig. 5A, five were blunt-end joins (microhomology or insertion of ≤1 bp), and two showed microhomology (≥2 bp). The remaining six junctions contained 2–20 bp of sequence with ambiguous origin. It is possible that these sequences have junctional microhomology that cannot be detected because the homologous sequences are derived from repeats and/or contain partial mismatches, making them difficult to map (46).

In light of these findings, we characterized the efficiency of DNA replication in chromosome bridges by pulse-labeling with the nucleoside analog, 5-ethynyl-2′-deoxyuridine (EdU). By contrast with primary nuclei, in S phase cells, EdU intensity dropped off in the bridge where it emerged from the main nucleus and was mostly absent from the remainder of the bridge (Fig. S11A). Likewise, broken bridge stubs also displayed defective DNA replication (Fig. S11A). Control experiments demonstrated that the absence of EdU signal was not simply a consequence of limited detection sensitivity for the small amount of DNA in bridges (Fig. S11B). Moreover, defective replication of bridge DNA could also be inferred from our single cell sequencing data (Fig. S11C). Thus, chromosome bridges exhibit severe DNA replication defects similar to those previously identified in micronuclei (12, 14, 17).

We observed the TST jump signature in two additional contexts, using different sequencing methods. First, we identified the TST jump signature by bulk sequencing of a population of cells derived from a single cell with a broken bridge. We induced the formation of CRISPR-generated Chr4 bridges, isolated individual cells after bridge breakage, and then grew each cell into a large population (>10^6^ cells). The TST jump signature, with 150 bp median insertion size, was identified in one of 12 populations (Fig. 5B and Fig. S10B), and another sample (sequenced to lower depth) displayed the characteristic breakpoint clustering. Second, we identified the TST jump signature in a tumor genome by single-molecule long-read sequencing of a primary tumor sample obtained from a patient with renal cell carcinoma. In this patient sample, the TST jumps are associated with a chromothripsis event between Chr3p and Chr5q (Fig. 5C). This unbalanced translocation is the single most common mechanism underlying Chr3p loss, the canonical driver event in this cancer type (47). Again, the size of the insertions (median 199 bp) in the tumor was similar to what we observed by single-cell sequencing of broken bridges (Fig. S10B). The TST jump signature therefore reflects a specific mutational process that can be stably inherited over many generations and is present in human primary tumors.

In summary, sequencing cells after the breakage of chromosome bridges demonstrates that most rearrangements result from ligation after localized fragmentation, but that highly complex rearrangements can occur in a minority of cases. The sequence features of these rearrangements (TST jumps) suggest an origin from template-switching errors in DNA replication (10, 46, 48, 49).

### Mechanisms generating DNA damage downstream of chromosome bridge breakage

#### Damage from aberrant mitotic DNA replication.

Although there is only a low frequency of complex rearrangement associated with the initial formation and breakage of chromosome bridges in the first generation, complex rearrangements appeared to arise frequently in the second generation (i.e. granddaughter cells, the progeny of daughter cells with broken chromosome bridges). In all three of the second-generation lineages examined by single-cell sequencing, we detected chromothripsis-like rearrangements localized near the bridge breakpoints (Fig. S12). This suggested that the broken stubs of chromosome bridges might acquire additional damage during passage through mitosis.

**Figure 6.**
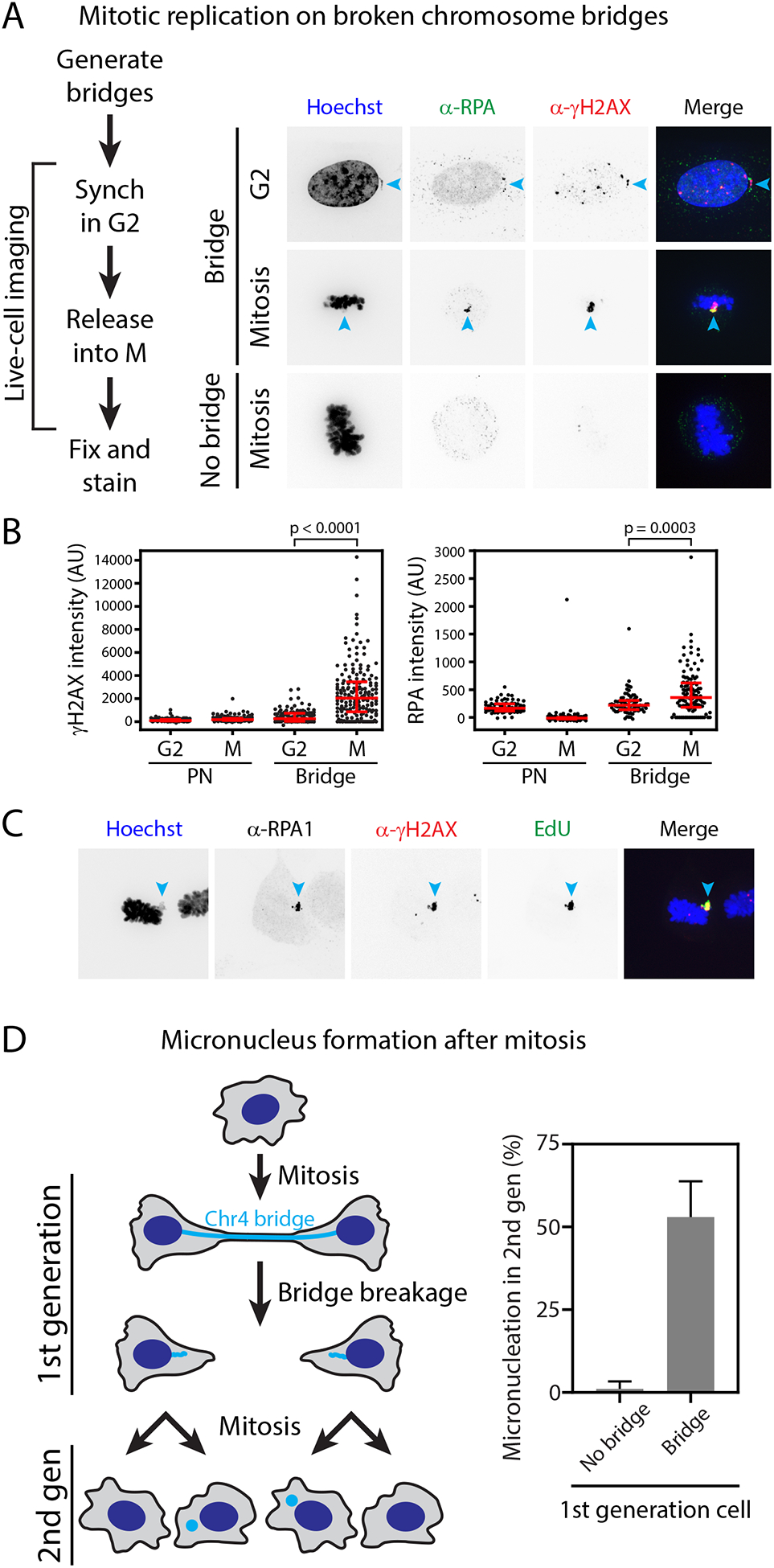
Broken bridge chromosomes undergo mitotic DNA damage and frequent mis-segregation to form micronuclei. (A) Mitosis-specific damage of bridge DNA detected by correlative live-cell/same-cell fixed imaging. **Left**: schematic of the experiment. **Right**: example images of cells with broken bridges in G2 or in mitosis, compared to a control mitotic cell (no bridge in the prior interphase). Cyan arrowheads: bridge chromosome. (B) Quantification from (A); *p*-values from Mann-Whitney test. (C) DNA damage (γ-H2AX) coincides with RPA accumulation and active DNA replication (EdU). Cyan arrowheads: bridge chromosome. (D) Frequent micronucleation in the second generation after bridge formation. **Left**: schematic of the live-cell imaging experiment. A cell divides, forming a CRISPR-induced Chr4 bridge (1^st^ generation). After bridge breakage, daughter cells divide, forming “granddaughter” cells (2^nd^ generation). **Right**: Frequency of micronucleation in 2^nd^-generation cells was measured for control cells that did not have a bridge in the 1^st^ generation (No bridge) as compared to cells that did (Bridge).

We assayed DNA damage in mitosis using a protocol of live-cell imaging followed by fixation and staining for γ-H2AX in these same cells. Relative to primary nuclei, most broken bridges exhibited little or no damage during interphase, even when cells were held in extended G2-arrest with cyclin-dependent kinase 1 (CDK1) inhibition. However, if cells with broken bridge stubs were released from G2-arrest into mitosis, γ-H2AX labeling intensity increased ~5-fold (Fig 6A–B). Heavy mitotic γ-H2AX labeling was consistently associated with extensive replication protein A (RPA) accumulation, indicating the generation of single-stranded DNA (ssDNA) (Fig. 6A–B and Fig. S13A). Surprisingly, pulse-labeling with EdU revealed that RPA and γ-H2AX accumulation coincided with extensive DNA synthesis that occurred specifically on the bridge DNA during mitosis (Fig. 6C). Similar findings were obtained in BJ cells (Fig. S13B). Live-cell imaging of GFP-RPA2 established that the mitotic replication was restricted to the stub of the broken chromosome bridge (Fig. S13A and Movie S7). Therefore, the stubs of broken chromosome bridges undergo a second wave of DNA damage during a burst of aberrant, mitosis-specific DNA replication.

#### Chromosome bridges generate micronuclei.

If chromosome bridge formation generated micronuclei, the frequency of chromothripsis and the size of the rearrangement footprint would be further increased (12, 15). This could contribute to the extensive pattern of rearrangements previously reported by bulk sequencing of cell clones derived after telomere crisis (28).

Although it was recently reported that micronuclei do not form immediately after chromosome bridge breakage (28), whether the resulting broken chromosomes segregate normally in subsequent cell divisions has not been examined. We therefore used live-cell imaging to track bridge chromosomes over two generations (Fig. 6D). Our imaging confirmed that micronucleation is not an immediate consequence of chromosome bridge breakage in the interphase during which the bridge forms and breaks. However, a different result was obtained when cells with broken bridges went through the next mitosis: 52% of divisions resulted in granddaughter cells with micronuclei (n=82 daughter cell divisions examined; Fig. 6D and Fig. S14A). When the bridge did not break during the first cell division, the frequency of micronucleation was higher still (65%, n=20, Fig. S14B). By comparison, cells without a bridge divided normally and did not produce micronuclei (n=82 divisions), even though they were present in the same imaging dish and were treated identically.

To determine whether the above-described micronuclei contain chromosomes from bridges, we induced CRISPR-generated Chr4 bridges and used fluorescence in situ hybridization (FISH) to detect DNA from Chr4. After induction, almost all bridges contained Chr4 sequence and in the second cell cycle, most micronuclei contained DNA from Chr4 (80%, n=105; Fig. S14C). Surprisingly, and inconsistent with early models (50), most of these micronuclei also contained Chr4 centromere DNA (62%, n=84), suggesting bridge formation and/or breakage disrupts centromere function. Thus, micronucleation is a major downstream consequence of chromosome bridge formation, regardless of whether the bridge breaks.

### Common mechanisms for DNA damage in micronuclei and chromosome bridges

Because micronuclei and chromosome bridges share a common nuclear envelope defect (17), we hypothesized that these structures, although differing morphologically, might nevertheless have a similarly defective nucleoplasm leading to defects in DNA replication—both during interphase and then later in mitosis. As a first step, we addressed whether micronuclei acquire replication-dependent DNA damage during interphase. Because nuclear envelope disruption itself causes DNA damage (16), we characterized micronuclei with intact nuclear envelopes, identified by the accumulation of nuclear-targeted red fluorescent protein (RFP-NLS) (16).

Micronuclei were generated by a nocodazole washout procedure (15), and EdU-labeling was used to assess the extent of DNA replication in micronuclei. Relative to the primary nucleus, many intact micronuclei in G2 cells showed detectable but strongly reduced DNA replication, as expected (median EdU ratio=27%). DNA damage was observed in a subset of intact micronuclei (23%), almost exclusively in micronuclei with the strongest replication defect (Fig. S15A–B), suggesting that DNA damage is coupled to defective replication. Furthermore, DNA damage was nearly eliminated by blocking the initiation of DNA replication with small molecule inhibitors of either cyclin-dependent kinase or Dbf4-dependent kinase (Fig. S15A–B). We note that while γ-H2AX intensity measurements were reliable for assessing DNA damage in micronuclei, similar measurements are not feasible for chromosome bridges, because of the tension-induced depletion of nucleosomes from stretched bridges (28). Importantly, single-cell sequencing showed extensive chromothripsis-like rearrangements in one of ten G2 cells with intact micronuclei (Fig. S15C). Thus, like chromosome bridges, intact micronuclei undergo defective DNA replication in interphase during the first cell cycle after their formation, which appears to generate a low frequency of DNA damage and chromothripsis.

We next determined whether micronuclear chromosomes, like broken chromosome bridges, undergo mitotic replication and secondary DNA damage. Although most intact micronuclei in G2 cells lacked DNA damage, after entering mitosis, there was a ~10-fold increase in damage levels on micronuclear chromosomes, accompanied by mitotic DNA synthesis and the extensive accumulation of ssDNA (Fig. S16 and Movie S8).

Single-cell sequencing demonstrated that transit through mitosis promotes chromothripsis of micronuclear chromosomes. By live-cell imaging, we identified cells with intact micronuclei that subsequently went through mitosis, generating daughter cells. By contrast with parental G2 cells where chromothripsis was rare (1/10 cells; Fig. S15C), chromothripsis was common in these daughter cells that had passed through mitosis (8/9 cell pairs, *p*=0.001, Fisher’s exact test; Fig. S16D). Thus, incompletely replicated chromosomes from either micronuclei or bridges undergo aberrant replication upon entry into mitosis, correlated with a high frequency of chromothripsis in the next generation.

In summary, at a low frequency, DNA from chromosome bridges or micronuclei undergoes fragmentation and rearrangement during defective DNA replication in interphase. Subsequently, a second wave of abnormal replication and heavy DNA damage occurs when cells enter mitosis. After mitosis, DNA damage and chromothripsis can be further amplified on bridge chromosomes by their frequent mis-segregation into micronuclei.

### Complex genome evolution from the formation of a chromosome bridge

The above-described findings predict that the formation of a chromosome bridge should initiate ongoing genome instability (51) where episodes of chromothripsis would necessarily occur at multiple steps of the breakage-fusion-bridge cycles (4, 22).

**Figure 7.**
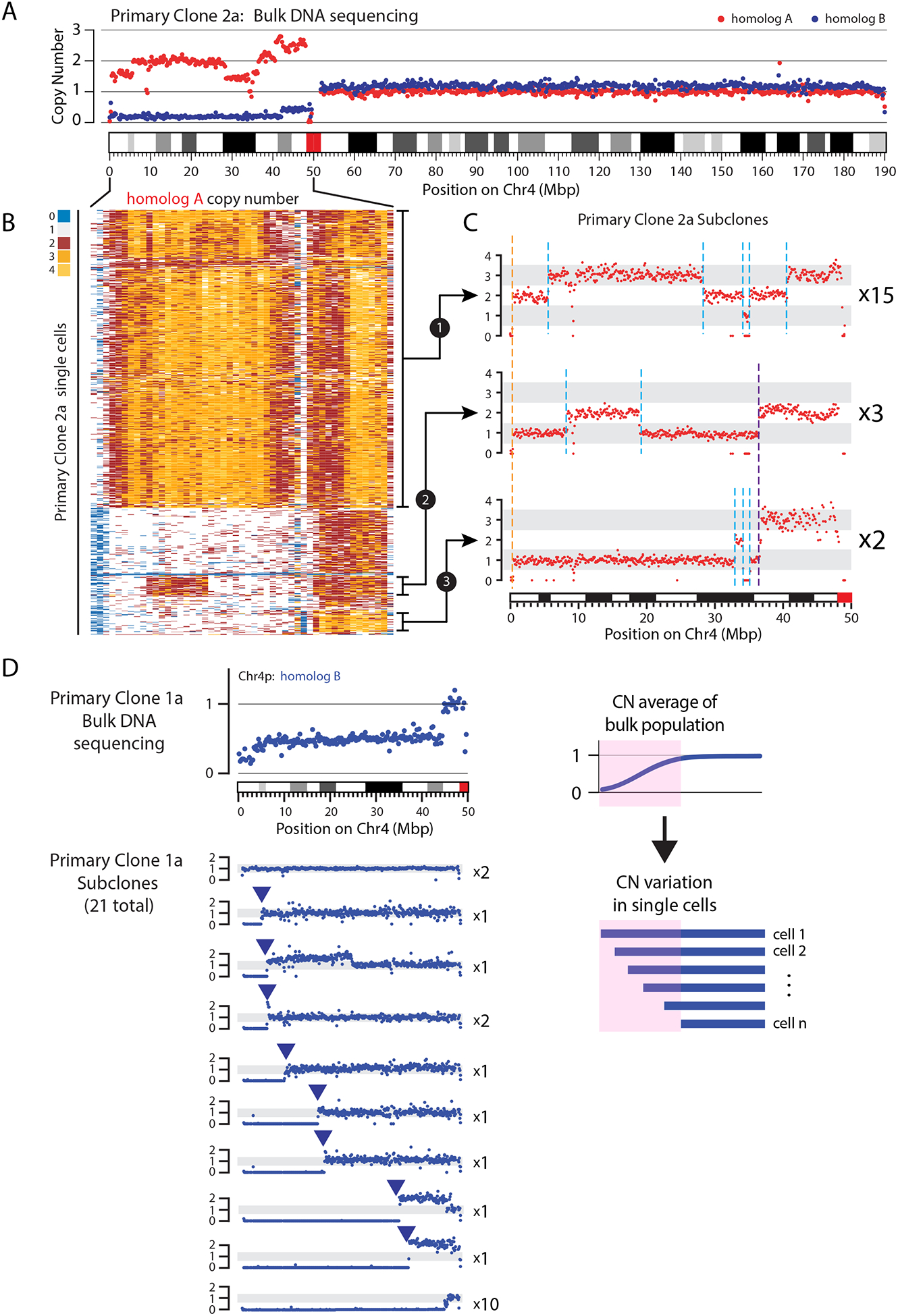
Ongoing instability and subclonal heterogeneity after chromosome bridge formation. (A) Bulk sequencing indicates subclonal heterogeneity within a primary clone derived from a single cell after bridge breakage. Plot shows DNA copy number (CN) for the two Chr4 homologs (red and blue dots, 25-kb bins). Regions of non-integer CN indicate the existence of subclones with different CN states. (B) CN heatmap for Chr4p (0–50 Mb) homolog A in 637 single cells. Each row represents one cell. Different subclonal populations can be identified that exhibit CN profiles consistent with those seen in single cell-derived subclones, shown in (C). (C) CN profiles for Chr4p homolog A (red dots, 25-kb bins) in 20 subclones grown from single cells isolated from one primary clone. One CN transition (breakpoint) is shared by all subclones (dashed orange line) whereas other CN changes are shared only among a subset of subclones (dashed purple line), or are private to individual subclones (dashed cyan lines). The number of subclones represented in each CN profile is listed next to each plot. (D) Detection of ongoing chromosomal instability in a primary clone. **Left Top**: CN for Chr4p homolog B from bulk sequencing of the primary clone. **Left Bottom**: 10 unique CN profiles identified from 21 single-cell derived subclones obtained from the primary clone. The number of subclones represented in each CN profile is listed next to each plot. **Right**: Schematic shows how gradual sloping copy number transitions in bulk populations are explained by subconal heterogeneity.

To test this model, we tracked the evolution of CRISPR-generated Chr4 bridges during long-term population growth. The parental line without CRISPR induction did not show alterations to Chr4 and maintained a stable karyotype (Table S2). By contrast, each of 12 clones isolated downstream of initial bridge formation and breakage (hereafter “primary clones”) contained an altered Chr4 based on cytogenetic analysis (Table S2). Additionally, bulk genome sequencing revealed copy number alterations that affected one or both homologs of Chr4 in each primary clone (Fig. 7A and Fig. S17).

In addition to the Chr4 aberrations, the primary clones had a total of 26 karyotype abnormalities affecting other chromosomes (Table S2). Nearly all non-Chr4 aberrations involved acrocentric chromosomes (85% of cases, Table S2), usually fused at their p-arms to an abnormal Chr4 (Fig. S18). This is unlikely to have resulted from off-target CRISPR cutting, because acrocentric fusions are also common in RPE-1 cells after TRF2-DN-mediated bridge induction (52). Acrocentric chromosomes may be frequently fused to other broken chromosomes because their p-arm rDNA repeats are fragile (53), or because fusion to an acrocentric chromosome is more likely to generate a single centromere. Importantly, the non-Chr4 aberrations were typically subclonal within each primary clone (Table S2 and Fig. S18), suggesting downstream evolution after breakage of the Chr4 bridge.

Ongoing genome instability within most of the primary clones was further supported by: (i) high frequencies of micronuclei and chromosome bridges and (ii) non-integer copy number states in the bulk sequencing data, indicating subclonal copy number heterogeneity (Fig. 7A and Fig. S19). Genetic heterogeneity between cells in the primary clones was directly verified by performing single-cell copy-number profiling (500–800 cells from each clone; Fig. 7B and Fig. S20). Extensive copy-number variation was observed, mostly confined to Chr4 but also on acrocentric Chrs 13, 14, 15, and 22 [Fig. 7B, Fig. S20, and see Dryad deposit (Data Materials and Availability)].

To better understand the evolution of copy number variation, we performed bulk whole-genome sequencing on subclones derived from single cells isolated from the primary clones (Fig. 7C–D). Analysis of these subclones provided clear evidence that complex chromosomal rearrangements occur downstream of bridge breakage.

First, in one set of subclones (derived from primary clone 2a) with copy number profiles exhibiting a single, shared ancestral breakpoint, we also identified additional breakpoints that occurred only within specific lineages (Fig. 7C). These breakpoints private to each lineage can only have been acquired after the shared ancestral break.

Second, in a different set of subclones (from primary clone 1a), we observed kataegis in 22 of 23 subclones; however, only a minority of these kataegis events were shared among all the subclones (Fig. S21). Most kataegis events were identified in only a subset of subclones or were private to just one subclone (Fig. S21), suggesting they arose late during population expansion.

Third, among this same set of subclones, we observed variation in both the location and the magnitude of focal amplifications on Chr4 homolog A (Fig. S22). BFB cycles are not conventionally considered to be mechanisms for internal-chromosome focal amplification, however, we suggest this could occur if bridge fragments are ligated to form extra-chromosomal circles (15).

Fourth, among nine subclones that shared a common copy-number profile of homolog A (Fig. S22, top profile), there was variable loss of homolog B from the p-arm terminus, a pattern suggestive of progressive shortening (Fig. 7D). These findings suggest that subclonal loss of homolog B occurred late during growth of the primary clone, postdating the alterations of homolog A. In this example, the apparent progressive shortening of homolog B likely reflects ongoing BFB cycles. The absence of cells with gain of this region, as expected from the original BFB model, could result from compromised fitness of cells with Chr4 terminal-segment gene amplification, and/or a bias towards segmental loss due to under-replication of bridge DNA (Fig. S11). This progressive terminal segment loss generates a characteristic gradual, sloping copy number transition in the bulk sequencing data (Fig. 7D). This pattern is apparent in several of our primary clones (Fig. S19) and has also been observed in cancer genomes (C.Z. Zhang, unpublished). We suggest this pattern may provide a useful bulk DNA sequence-based biomarker of ongoing genome instability.

## DISCUSSION

Our results identify a cascade of events that generate increasing amounts of chromothripsis after the formation of a chromosome bridge, creating many hallmark features of cancer genomes from a single cell division error. We demonstrate that episodes of chromothripsis will be inherently interwoven with multiple steps of the BFB cycle. This motivates a substantial revision of the chromosome breakage-fusion-bridge model (18, 19, 54) that explains the inferred association between these processes in cancer genomes.

We propose the following model (Fig. S23). Like micronuclei, nuclear envelope assembly around chromosome bridges is aberrant, leading to depletion of nuclear pores (4, 17, 50), which combined with bridge geometry (55), leads to a defective nucleoplasm. This results in poor DNA replication in the bridge, producing stalled replication forks and replication origins that have not fired. The bridge is then broken by a mechanism that requires stretching force from the actin cytoskeleton. Bridge breakage produces simple breaks and local fragmentation, generating free DNA ends that can engage in end-joining and/or in error-prone replicative repair, potentially MMBIR (10, 45). In some cells, this produces the rearrangement signature that we term Tandem Short Template (TST) jumps. These events lead to a low frequency of chromothripsis during the interphase when the bridge forms and breaks. Subsequently, after cells enter mitosis, the stubs of broken chromosome bridges undergo a burst of aberrant mitotic DNA replication, similar to what occurs for micronuclear chromosomes. This leads to significantly more DNA damage and increases the frequency of chromothripsis. Finally, bridge formation compromises centromere function, which increases the rate of micronucleation during the next cell division after bridge formation. These micronuclei will generate further cycles of chromothripsis, as previously described (12, 13, 15). Combined, these mutational events rapidly generate hallmark features of cancer genome complexity, producing continuing cycles of genome evolution and ongoing subclonal heterogeneity from a single cell division error.

### Mutagenesis and DNA fragmentation from actomyosin-based force

It was previously proposed that bridge breakage might occur by mechanical forces generated during chromosome segregation in mitosis (19), cytokinetic furrow ingression, or abscission (24, 25). However, it now appears that most bulky chromosome bridges are only broken after these events, during interphase (28). Interphase bridges were suggested to be cleaved enzymatically via a mechanism partially dependent upon the cytoplasmic exonuclease TREX1 (28). However, our data disfavor a role for TREX1 and, instead, demonstrate that bridge breakage requires mechanical forces from the interphase actin cytoskeleton (Fig. 1). These forces appear to be exerted locally on DNA near the base of the bridge and are associated with transient actomyosin accumulation and large focal adhesions. Actomyosin forces appear to be transmitted in part across the nuclear envelope to the bridge chromatin by the LINC complex (56, 57).

A simple interpretation of our results is that actomyosin-dependent forces are capable of rupturing the phosphodiester bonds in bridge DNA. The force required to break DNA (58, 59) is estimated to be almost an order of magnitude lower than traction forces generated from individual focal adhesions (38, 60–62). Although non-covalent interactions connecting actin to chromatin are expected to be individually weak, large numbers of attachments acting in parallel could support the high mechanical load needed to break DNA. It is also possible that bridge breakage involves DNA processing enzyme(s) whose activity or access to DNA is enhanced by mechanical tension. Additionally, actomyosin-mediated disruption of nuclear envelope integrity could enable access of cytoplasmic nucleases to bridge DNA. However, we did not detect an impact of nuclear envelope rupture on bridge breakage, which generally disfavors a mechanism based on NE-restricted access of cytoplasmic nucleases to bridge DNA. We therefore propose that mechanical force is either sufficient for DNA breakage or facilitates the action of one or more nuclear-localized factors, such as a nuclease or topoisomerase.

Single-cell sequencing after chromosome bridge breakage identified either simple breaks or local DNA fragmentation, consistent with a breakage mechanism involving mechanical force. Importantly, we also observed both simple breakage and fragmentation when we mechanically broke intact chromosome bridges with a glass capillary. In principle, mechanical bridge breakage could cause localized chromosome fragmentation if forces were applied to multiple sites on chromatin, as might occur if chromatin were in a looped conformation.

### Chromosomal rearrangements from abnormal nuclear architecture

When bridge breakage was accompanied by fragmentation, we often observed chromosome rearrangements consistent with fragment re-ligation. Depending on the degree of fragmentation, this generated a range of outcomes (Fig. 3), from simpler patterns similar to the “local jump” footprint described in cancer genomes (42), to more complex events meeting conventional criteria for chromothripsis (63) (Supplemental Text).

A subset of bridge breakage events (4 of 20) showed a distinct pattern of extreme localized rearrangements, where small (~1–10 kb) regions contained focal clusters of ~10 breakpoints each. These “hotspots” were extensively inter-connected by rearrangements, despite being situated megabases apart in the reference genome or, occasionally, on different chromosomes. This generated a signature of multiple short (~200 bp) insertions present in tandem within rearrangement junctions (TST jumps; Fig. 5). We think TST jumps are likely generated by aberrant DNA replication involving replication template switching (10) for the following reasons. First, local breakpoint clusters are not expected from a random fragmentation process but could be generated by localized cycles of replication fork collapse, breakage, and error-prone replicative repair. Second, the size distribution of inserted segments (Fig. S10B) is inconsistent with random fragmentation and re-ligation. Consistent with micronuclei and chromosome bridges having similar functional defects, we previously identified an example of multiple short tandem insertions in single-cell analysis of chromothripsis derived from a micronucleus (15).

The TST jump signature does not result from artifacts during single-cell whole-genome amplification because a similar pattern was observed in bulk sequencing analysis of clonal populations of cells after bridge breakage. Furthermore, we observed a similar signature by single-molecule long-read sequencing of a renal cell carcinoma genome. Features of the TST jump signature have been noted in a variety of other contexts (42, 64, 65), although never previously fully defined, including lung cancer and in populations of cells deficient in non-homologous end-joining that emerged from telomere crisis (66), indicating that TST jumps may be common in cancer genomes. Although the cause of the TST jump signature is unknown, the restricted size distribution of the insertions might be generated by a low-processivity DNA polymerase, or possibly by the use of Okazaki fragments as replication templates.

We observed similar DNA replication abnormalities occurring in chromosome bridges and intact micronuclei. This makes sense as both nuclear structures have a similar defect in nuclear envelope assembly (17), which should generate a similarly defective nucleoplasm. In general, DNA replication errors are thought to be major sources of structural variation in cancer genomes. However, what triggers these severe replication abnormalities in the first place remains poorly understood. We propose that nuclear architecture defects, a hallmark feature of cancer termed nuclear atypia (67), are a major trigger for cancer-associated DNA replication errors.

### A second wave of DNA damage from aberrant mitotic DNA replication

We uncovered an unexpected burst of DNA replication that occurs during mitosis, specifically on the stubs of broken chromosome bridges or on micronuclear chromosomes. By contrast with a previously reported form of mitotic replication that is proposed to be beneficial for cells (68), the mitotic DNA replication described in our study is highly aberrant and produces heavy DNA damage and ssDNA formation.

The mechanism triggering mitotic DNA replication on bridge stubs or micronuclear chromosomes is not known. However, because bridge and micronuclear DNA is incompletely replicated during interphase, these structures likely contain stalled DNA replication forks and licensed replication origins that have not fired. We previously found that incomplete DNA replication in micronuclei occurs because of defective nucleocytoplasmic transport, leading to a failure to accumulate key proteins required for DNA replication and repair (4, 12, 17). However, when cells enter mitosis, the nuclear envelope is broken down, and under-replicated bridge or micronuclear DNA will suddenly gain access to the pool of replication factors that were sequestered in the primary nucleus throughout interphase. Access to replication factors, coupled with high mitotic cyclin-dependent kinase activity (69), likely then triggers mitotic replication of this incompletely replicated DNA. The DNA damage correlated with mitotic DNA replication may have a number of causes, including the well-described activation of structure-specific endonucleases in mitosis (70) and/or the recently discovered cleavage of stalled DNA replication forks that occurs because of removal of the MCM2–7 replicative helicase from mitotic chromosomes (71, 72).

### Chromosome bridges generate micronuclei

We found that chromosome bridge formation predisposes to micronucleation, which could then initiate additional rounds of chromothripsis downstream of bridge breakage (12, 15, 73). Because bridge breakage usually generates micronuclei with a centromere-containing chromosome fragment, it appears that bridge formation or breakage compromises centromere/kinetochore function. The mechanism for this centromere inactivation remains an interesting open question. Because stretching of chromosome bridges causes histone ejection (28, 74), we speculate that actomyosin forces could also strip CENP-A-containing nucleosomes when centromeric chromatin is trapped within the bridge. Thus, in addition to promoting mutagenesis, actomyosin contractility may disrupt epigenetic marks on chromatin.

### Rapid genome evolution from a single cell division error

The above-described cascade of events is predicted to generate ongoing cycles of complex genome evolution, a hypothesis that we tested with a CRISPR-based system to track the fate of a defined chromosome bridge over many generations. In these populations, we detected extensive genetic heterogeneity, with evidence that complex rearrangement continually recurs downstream of bridge breakage.

Together, our findings identify mechanisms that explain the remarkable potential of a single unrepaired DNA break to compromise the integrity of the genome. In human cells, a single DNA break has little capacity to activate the DNA damage checkpoint or cause cell cycle arrest (75, 76). An unrepaired break can therefore lead to many additional breaks due to the generation of micronuclei or additional chromosome bridges after cell division. Because de novo telomere addition is inefficient (77), stable end-capping of chromosomes is primarily achieved through chromosome translocation or break-induced DNA replication (78). An additional constraint is that the rearranged chromosome must contain only one functional centromere. The end result is that downstream of chromosome bridge formation, the accumulating burden of DNA breakage can easily exceed the capacity to stabilize broken chromosome ends. Therefore, complex genome evolution with subclonal heterogeneity is virtually an inevitable consequence of chromosome bridge formation, itself a common outcome of cell division defects during tumorigenesis.

## METHODS SUMMARY

Cell culture, drug treatments, and imaging were performed essentially as described (15) (see Supplemental Methods for details). Look-seq experiments were performed as described (15), with the exception that a CellEctor system (Molecular Machines & Industries) was employed in most cases for cell isolation. For long-term evolution experiments, the Look-Seq procedure was used with the following modifications. After bridge breakage, single cells were isolated into 96-well culture plates and then grown into large populations (<10^6^ cells each). Cells were then taken from the populations for karyotyping, bulk sequencing, and single-cell copy number analysis with the Chromium kit (10X Genomics). In some cases, single cells from populations were flow-sorted into 96-well culture plates for subcloning, followed by bulk sequencing. Single-cell genome amplification, sequencing library construction, and whole-genome sequencing were done as described (15), except that most sequencing was done on the NovaSeq platform.

## Supplementary Material

Supplementary Material (Supplementary Figures, Tables and Methods)

Movie S1

Movie S3

Movie S4

Movie S2

Movie S5

Movie S6

Movie S8

Movie S7
